# Supplementary material for: Bioassay-Guided Isolation of Two Eudesmane Sesquiterpenes from Lindera strychnifolia Using Centrifugal Partition Chromatography
Source: Molecules. 2021 Aug 30;26(17):5269. doi: 10.3390/molecules26175269 (PMC8433645; doi:10.3390/molecules26175269)
Supplement: Supplementary file 1 [file molecules-26-05269-s001.zip › molecules-1348660-supplementary.pdf]

*Supplementary Material*

# Bioassay-Guided Isolation of Two Eudesmane Sesquiterpenes from *Lindera strychnifolia* Using Centrifugal Partition Chromatography

**Ji Hoon Kim [1,†], Je-Seung Jeon [1,†], Jung Hoon Kim [1], Eun Ju Jung [1], Yun Jung Lee [1], En Mei Gao [1], Ahmed Shah Syed [2], Rak Ho Son [1,3] and Chul Young Kim [1,*]**

[1] College of Pharmacy and Institute of Pharmaceutical Science and Technology, Hanyang University, Ansan 15588, Korea; gg890718@gmail.com (J.H.K.); jsjeoncy@gmial.com (J.-S.J.); evveje@naver.com (J.H.K.); jejs2@naver.com (E.J.J.); sopihya@naver.com (Y.J.L.); rhdmsal@hanyang.ac.kr (E.M.G.); sonnaco@huons.com (R.H.S.)

[2] Department of Pharmacognosy, Faculty of Pharmacy, University of Sindh, Jamshoro 76080, Pakistan; shahahmed454@gmail.com

[3] R&D Center, Huons Co. Ltd., Ansan 15588, Korea

* Correspondence: chulykim@hanyang.ac.kr; Tel.: +82-31-400-5809

† These authors contributed equally to this work.

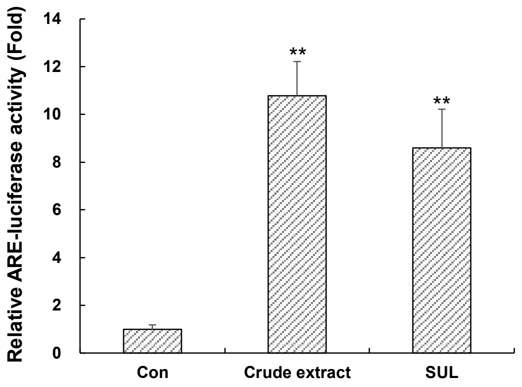


**Figure S1.** The relative ARE-luciferase activity of *Lindera strychnifolia* extract. ARE induction activity was evaluated in ARE-HepG2 cells at concentration of 30 μg/mL. Sulforaphane (SUL, 5μM) was used as positive control. Data are presented as means ± S.E. (n = 3). **, P < 0.01 (compared with the vehicle-treated control).

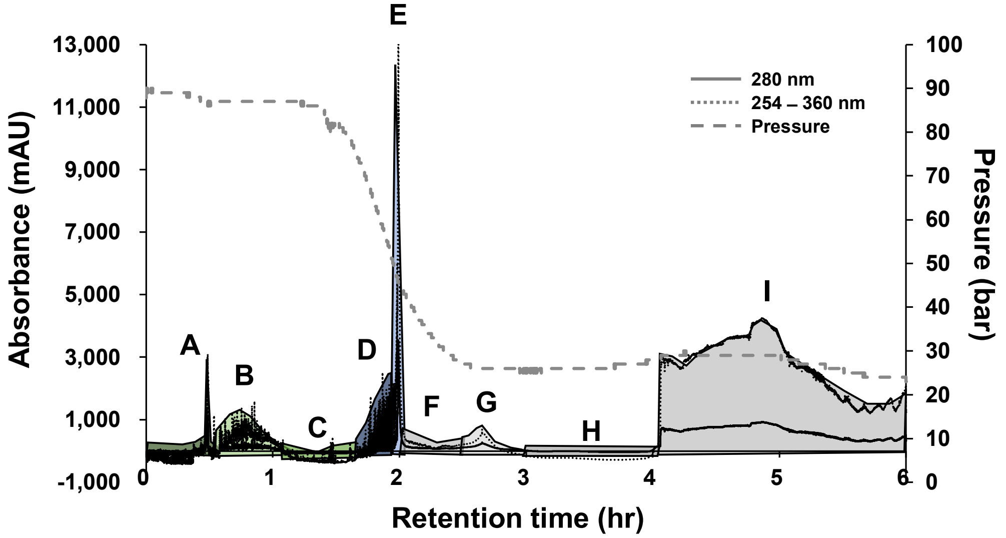


**Figure S2.** CPC chromatogram of crude *L. strychnifolia* extract. CPC operation condition was as follows; The lower layer of n-hexane–acetonitrile–water (10:2:8, v/v/v) was used as the stationary phase and upper layer of A (n-hexane–acetonitrile–water, 10:2:8, v/v/v), B (upper layer of ethyl acetate–acetonitrile–water, 10:2:8, v/v/v), and C (upper layer of water-saturated *n*-butanol–acetonitrile–water, 10:2:8, v/v/v) were subsequently eluted as mobile phase with a flow rate of 10 mL/min. Step gradient elution of mobile phase was as follows: 0–120 min (100% A), 120–180 min (100% A–100% B), 180–240 min (100% B), 240–300 min (100% B–100% C), and 300–360 min (100% C). The rotation speed of the rotor was 900 rpm. Following that, all the remaining samples were recovered by elution of methanol at 50 mL/min. One gram of crude sample was dissolved in 20 mL of mixed upper and lower phases and subjected to the CPC system.

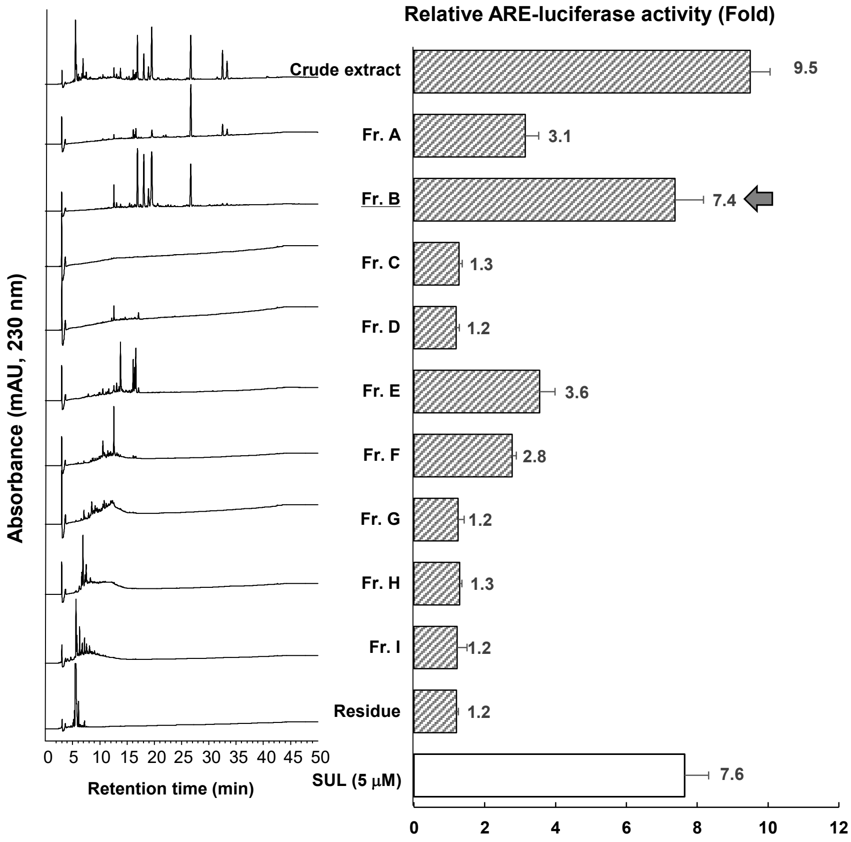


**Figure S3.** HPLC chromatograms and relative ARE-luciferase activity of CPC-fractions (**A**–**R**). Each CPC fraction was analyzed by HPLC, and its ARE induction activity was evaluated in ARE-HepG2 cells at concentration applied at each assigned weight ratio (based on 30 μg/ml CME). Data are presented as means ± S.E. (n = 3).

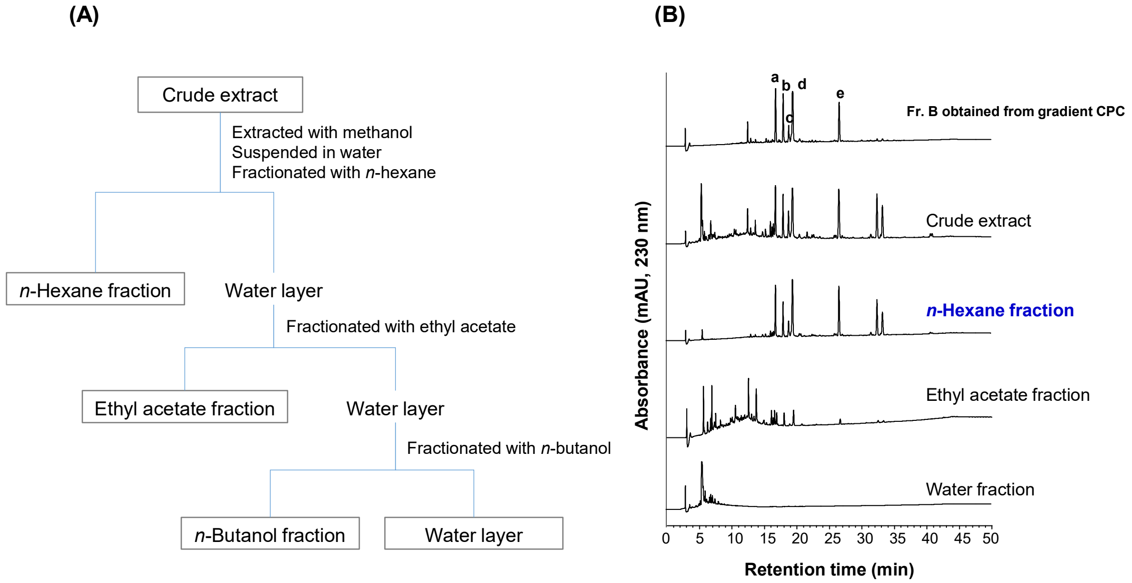


**Figure S4.** Comparison of solvent fraction and Fr. B obtained from gradient CPC. Scheme of solvent fractionation (**A**) and HPLC analysis of Fr. B (active fraction) and solvent extracts (**B**). HPLC conditions are as follows; column: Capcellpak UG120 C18 column (4.6 × 250 mm, 5 μm, Shiseido, Tokyo, Japan), mobile phase: acetonitrile containing 0.1% formic acid (**A**) and water containing 0.1% formic acid (**B**), gradient elution: 0–10 min, 10–50 % A; 10–45 min, 50–95% A; and 50 min, 95% A, column temperature: 40°C, mobile phase flow rate: 1 mL/min, the detection wavelength: 230 nm, and the injection volume: 10 μL.

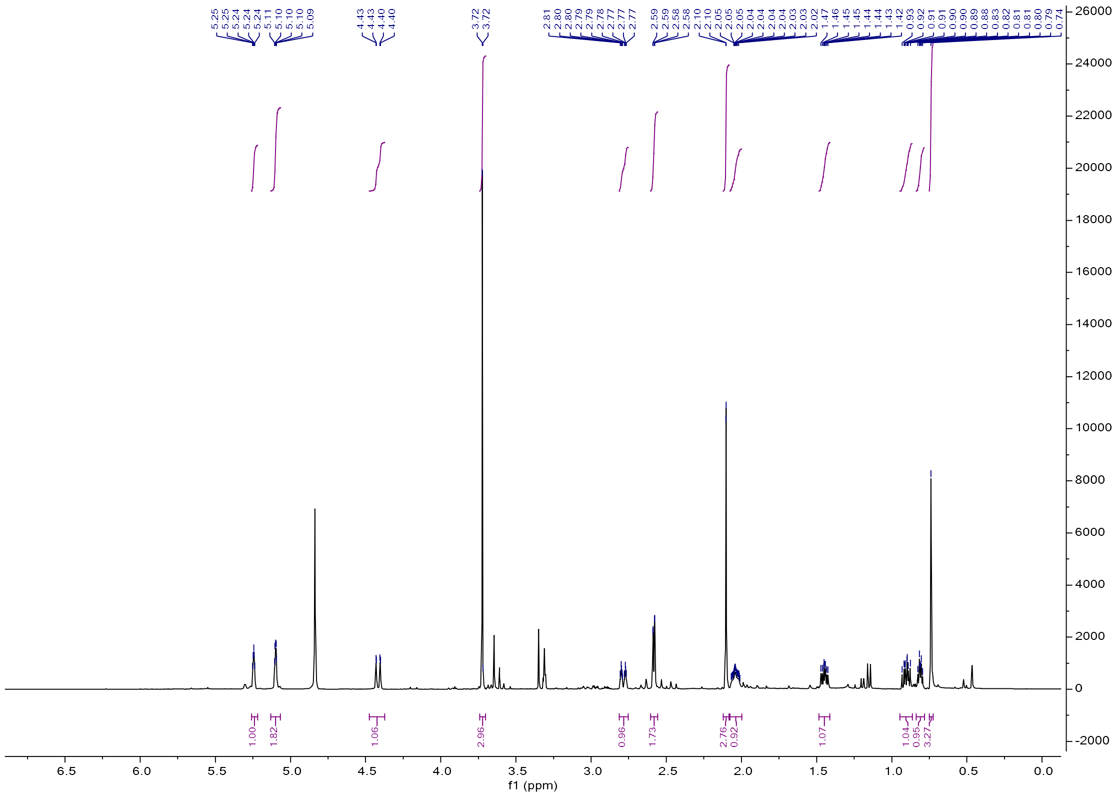

**Figure S5.** $^{1}H$ NMR spectrum of **1** (400 MHz) in $CD_3OD$.

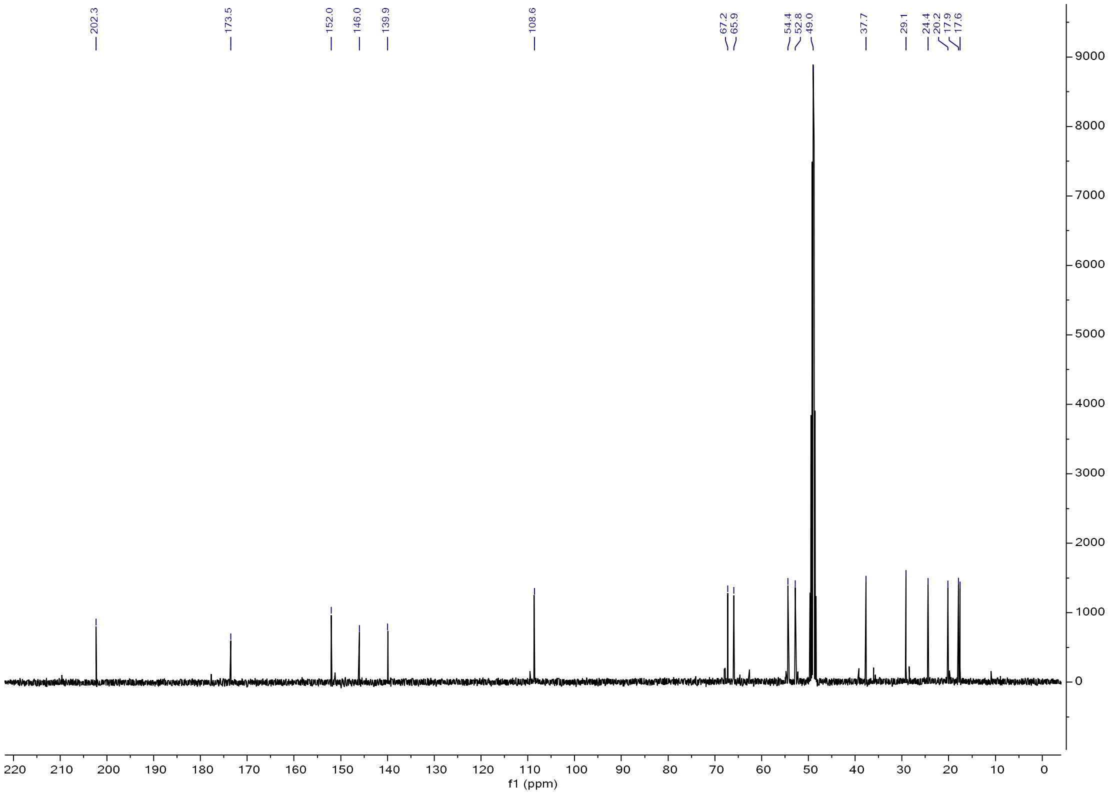


**Figure S6.** $^{13}$C NMR spectrum of **1** (100 MHz) in $CD_3OD$.

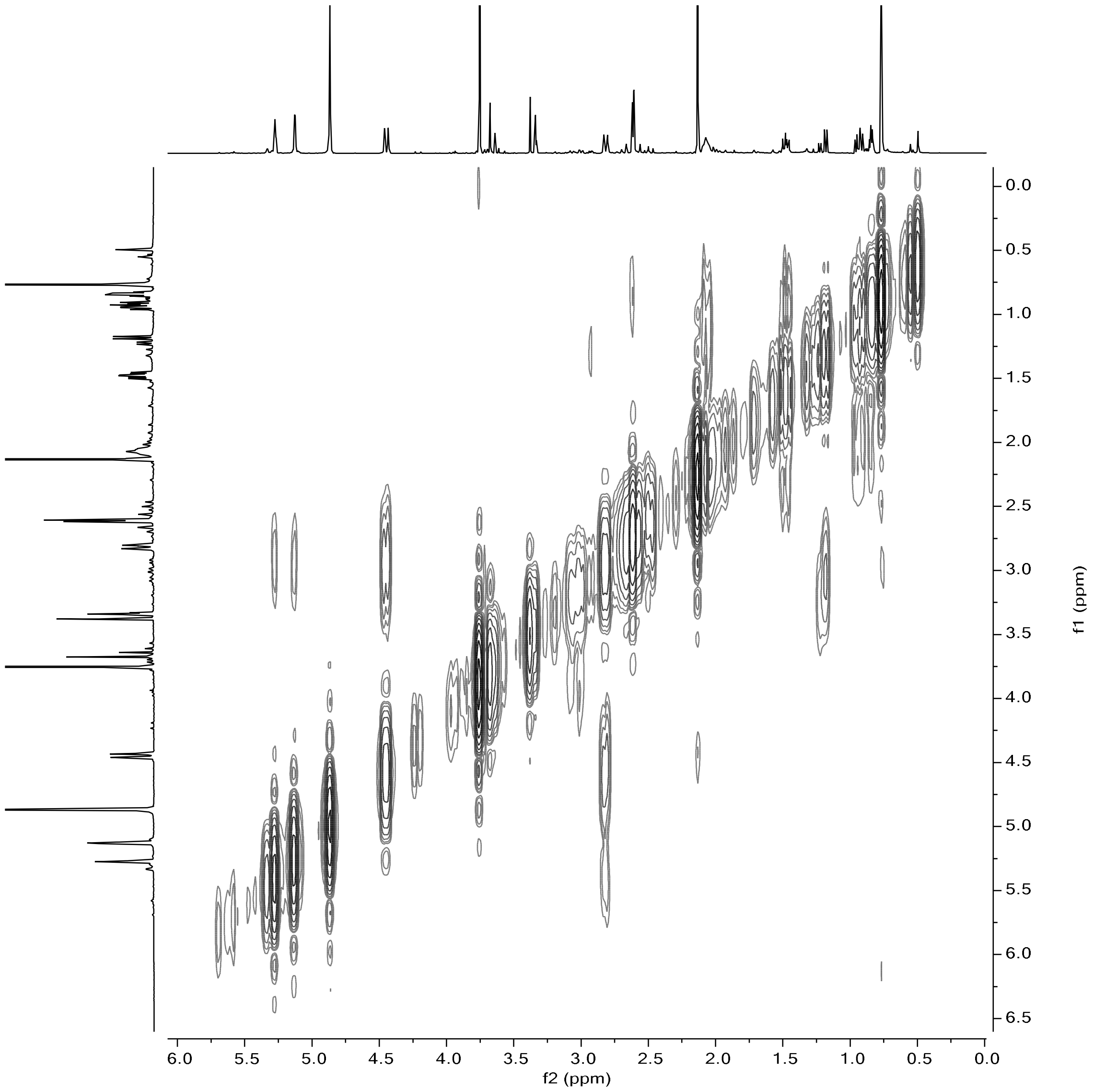


**Figure S7.** $^{1}H$-$^{1}H$ COSY spectrum of **1** in $CD_3OD$.

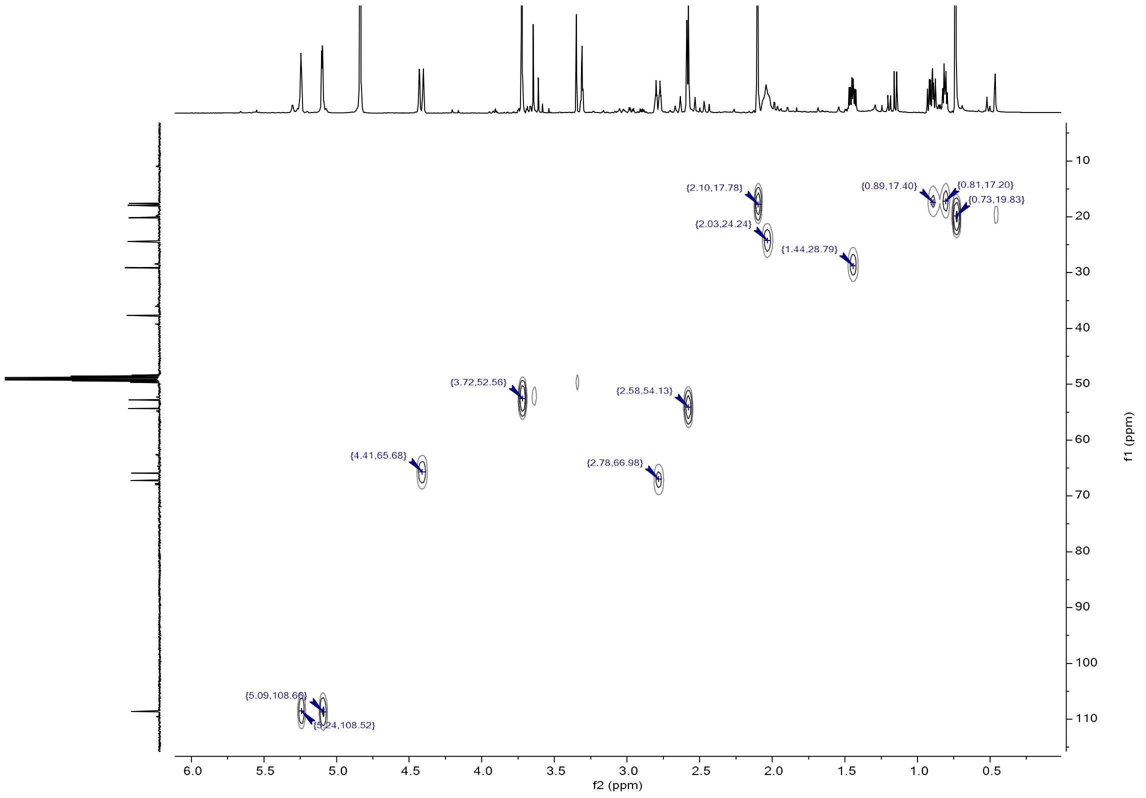


**Figure S8.** HSQC spectrum of **1** in $CD_3OD$.

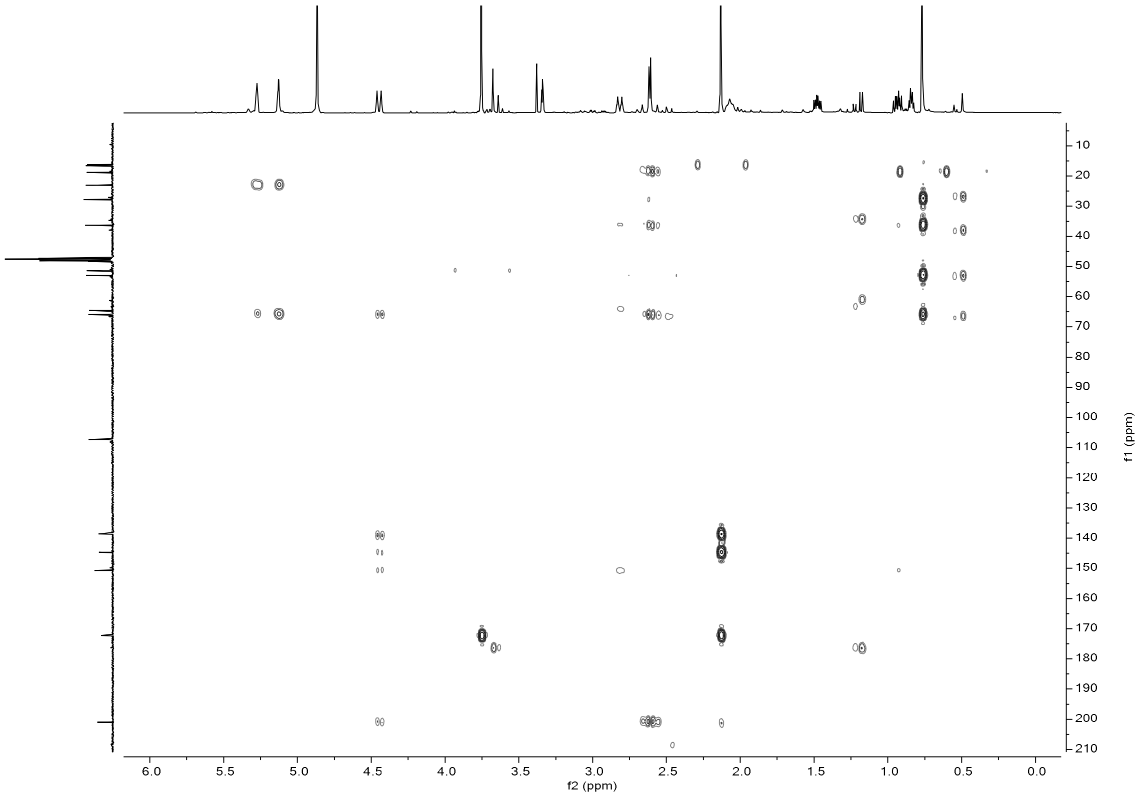


**Figure S9.** HMBC spectrum of **1** in $CD_3OD$.

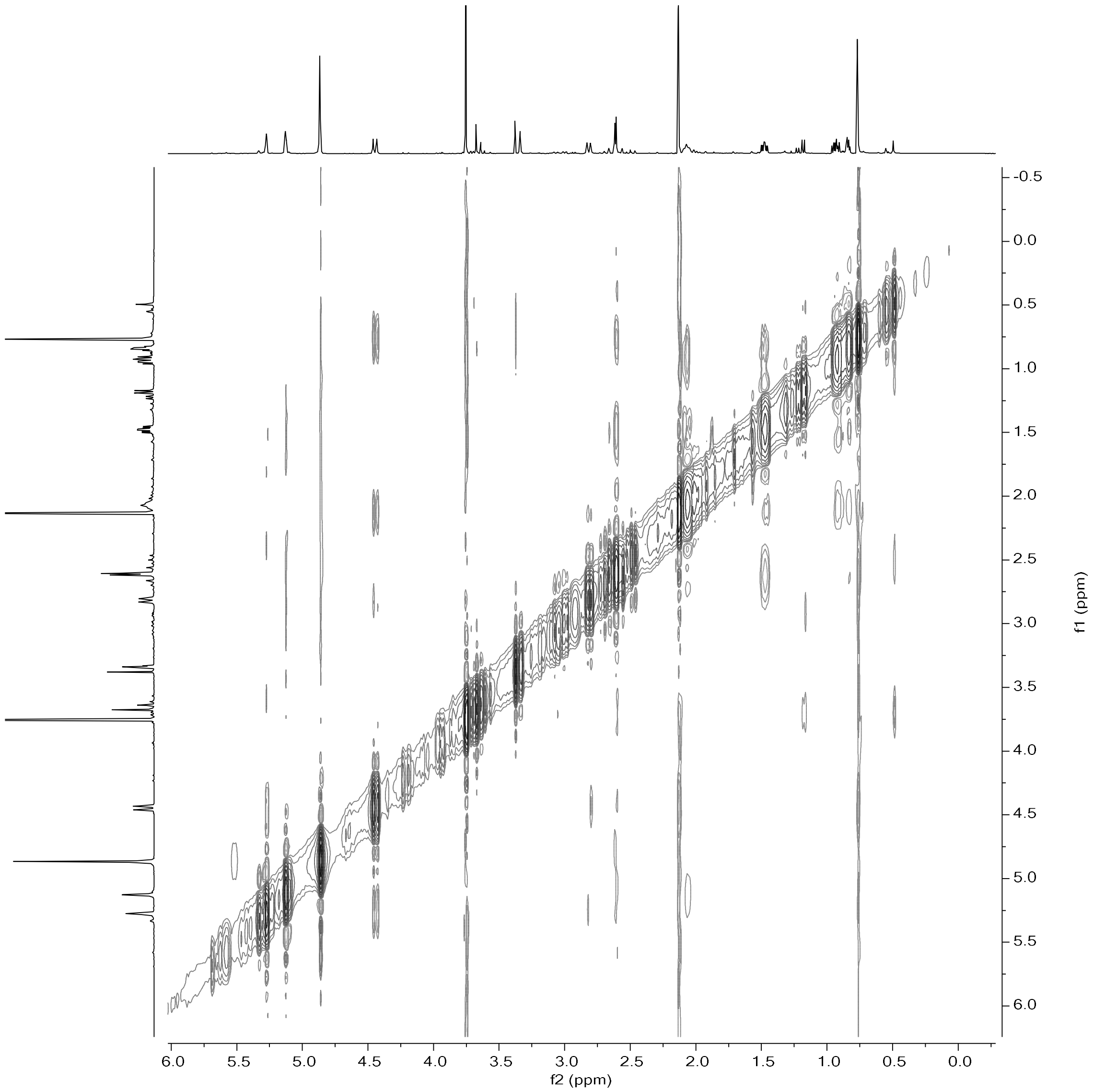


**Figure S10.** NOESY spectrum of **1** in $CD_3OD$.

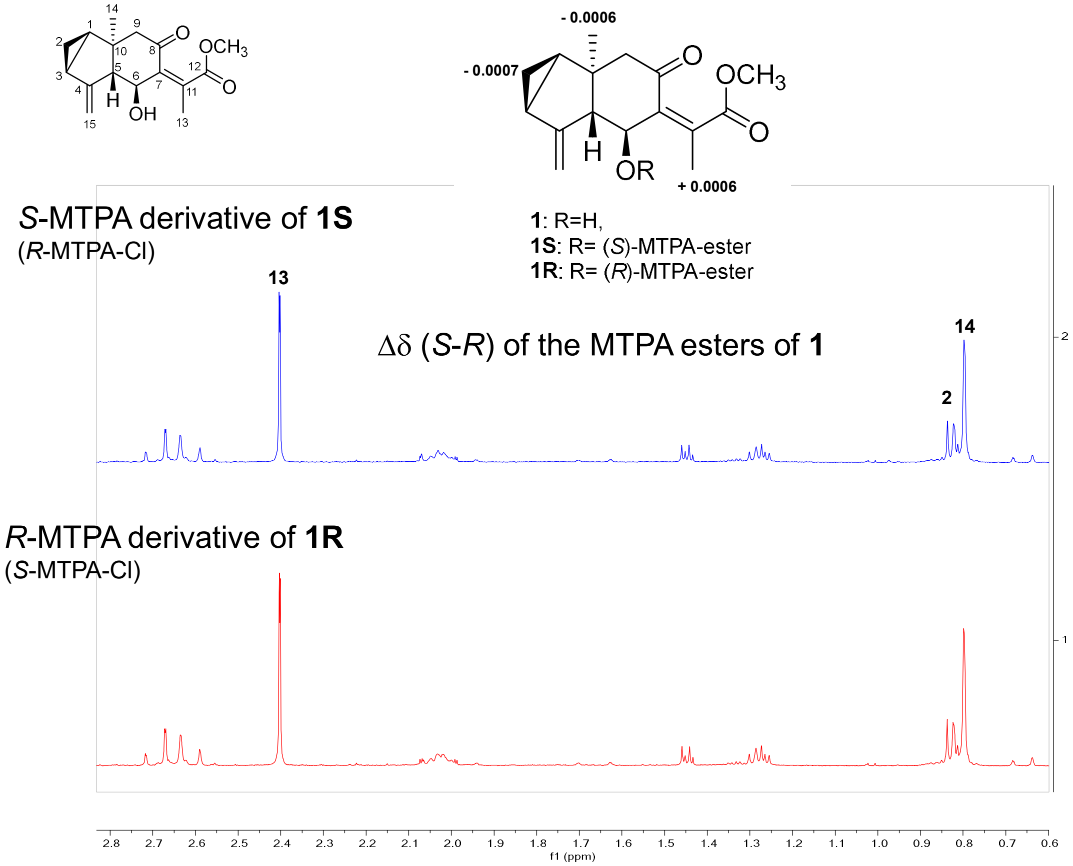


**Figure S11**. $^1$H NMR spectra of MTPA esters **1S** and **1R** and $\Delta\delta(S\text{-}R)$ values for (*S*)- and (*R*)-MPTA esters (**1S** and **1R**).

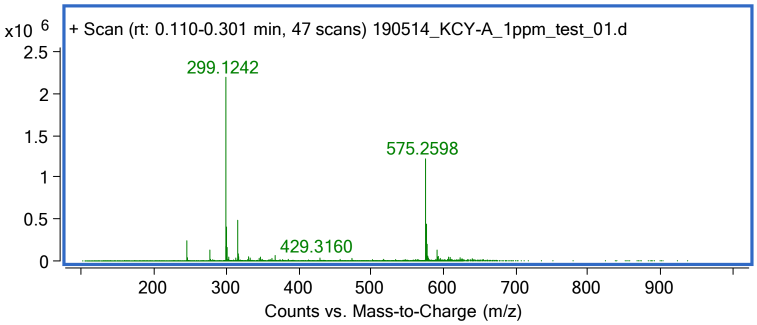


**Figure S12**. HRESI (qTOF) mass spectrum of **1**.

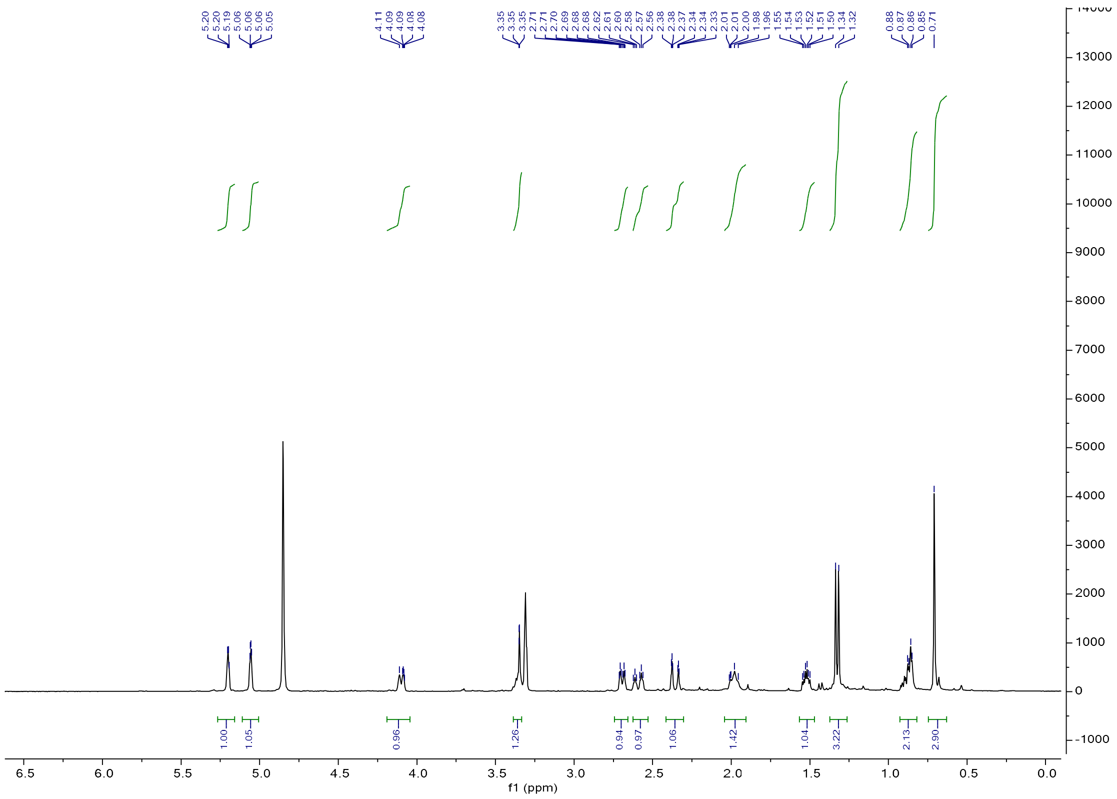

**Figure S13.** $^{1}H$ NMR spectrum of **2** (400 MHz) in $CD_3OD$.

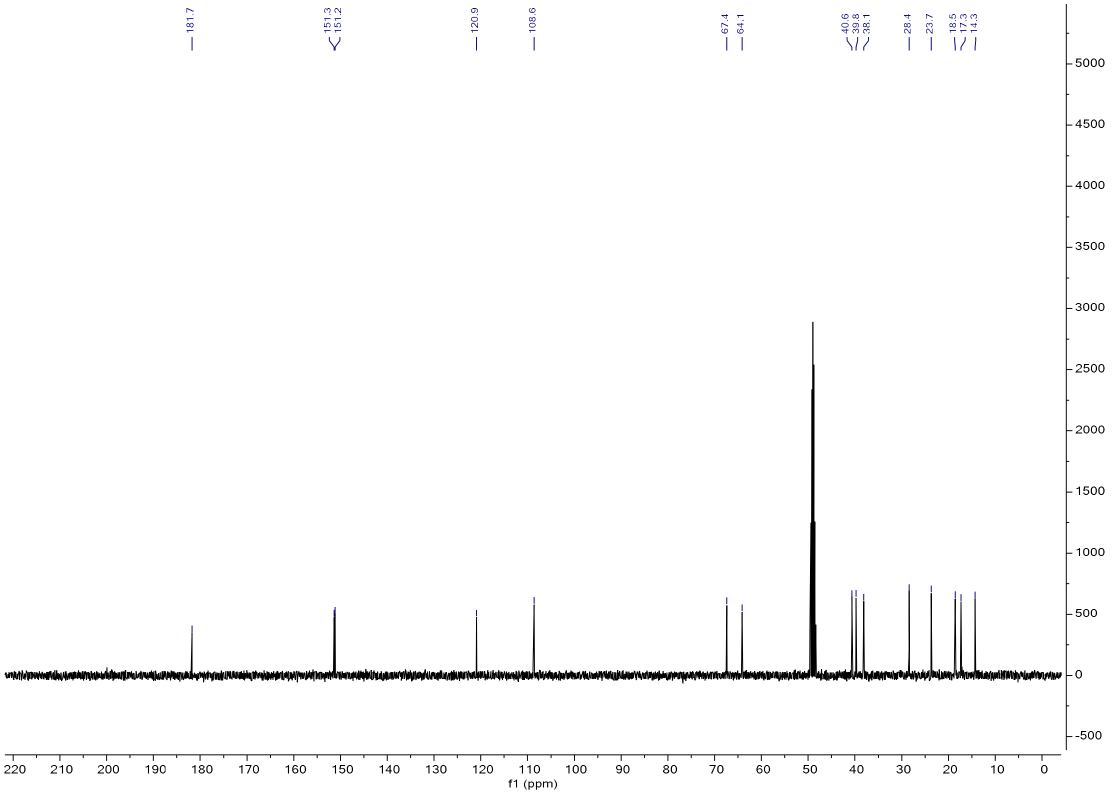


**Figure S14.** $^{13}C$ NMR spectrum of **2** (100 MHz) in $CD_3OD$.

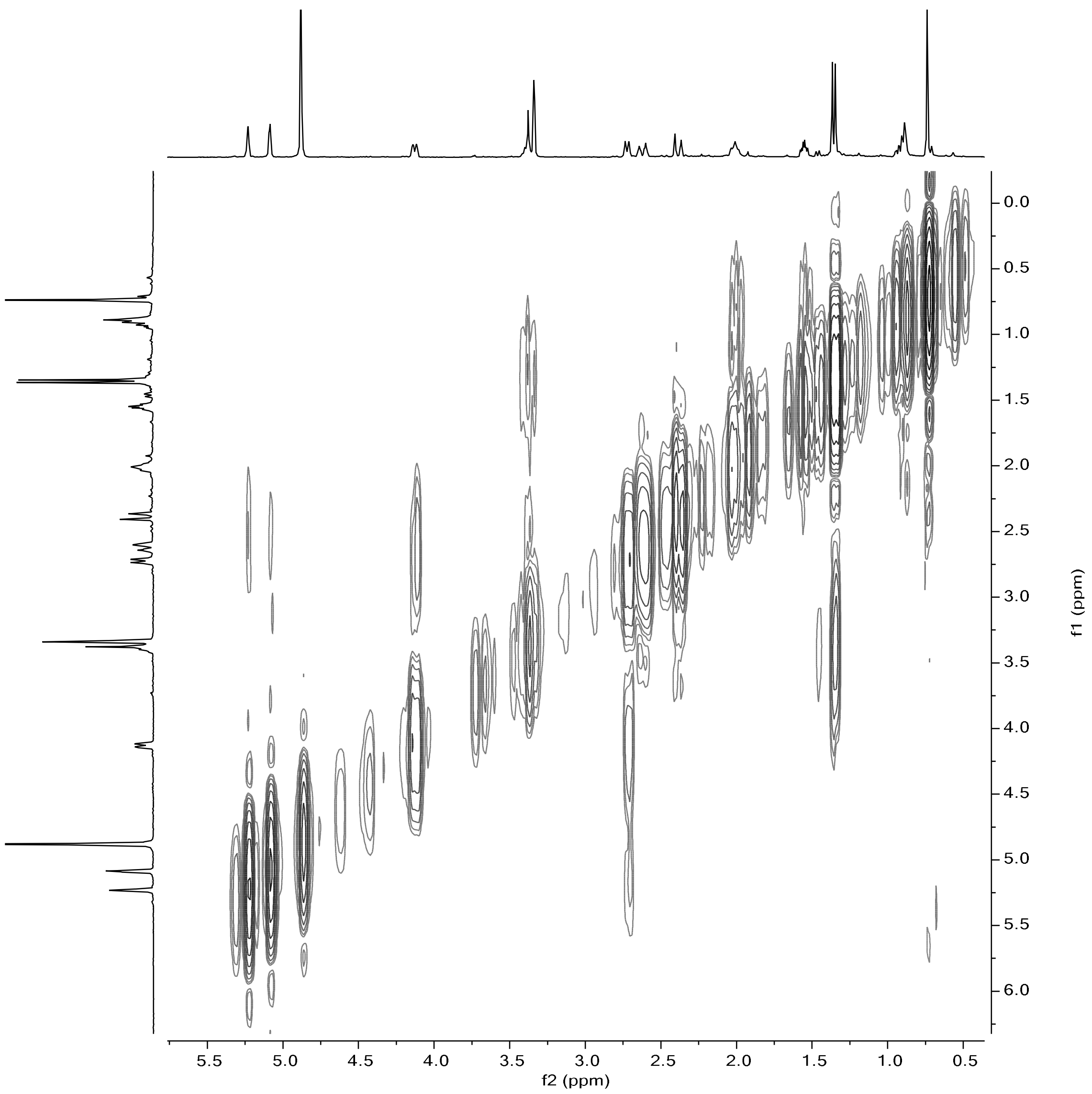


**Figure S15.** $^1H$-$^1H$ COSY spectrum of **2** in $CD_3OD$.

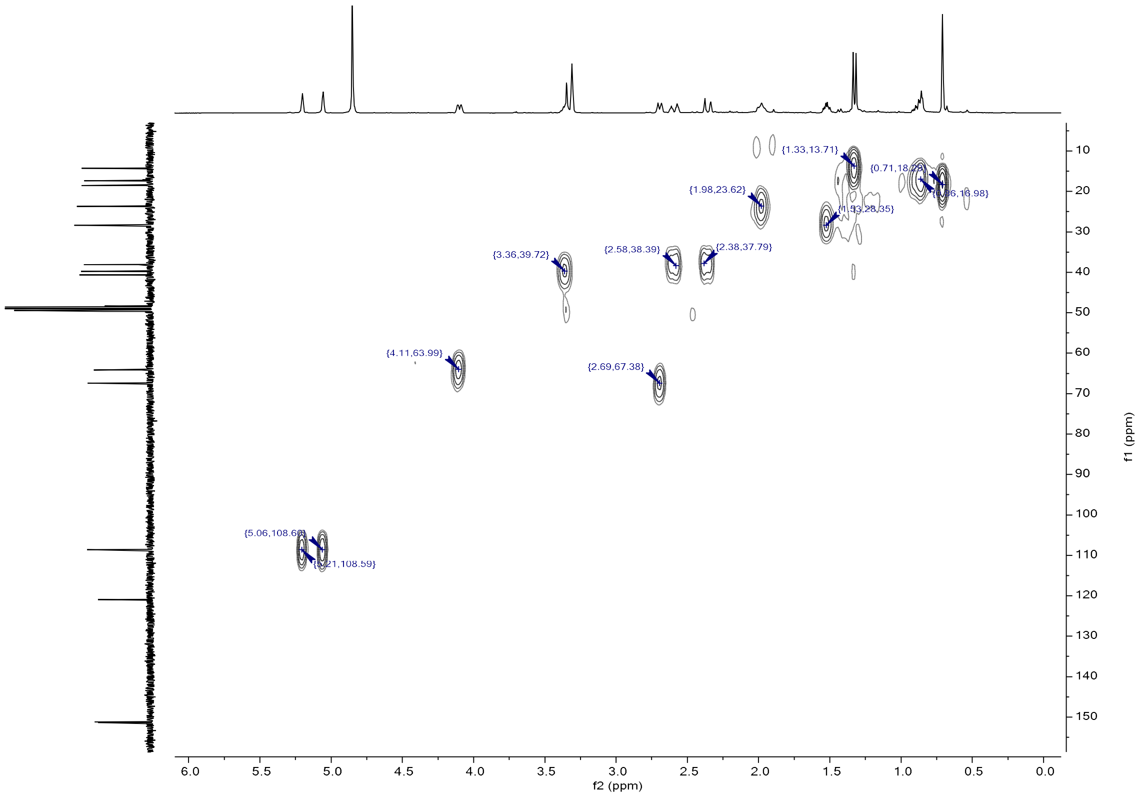


**Figure S16.** HSQC spectrum of **2** in $CD_3OD$.

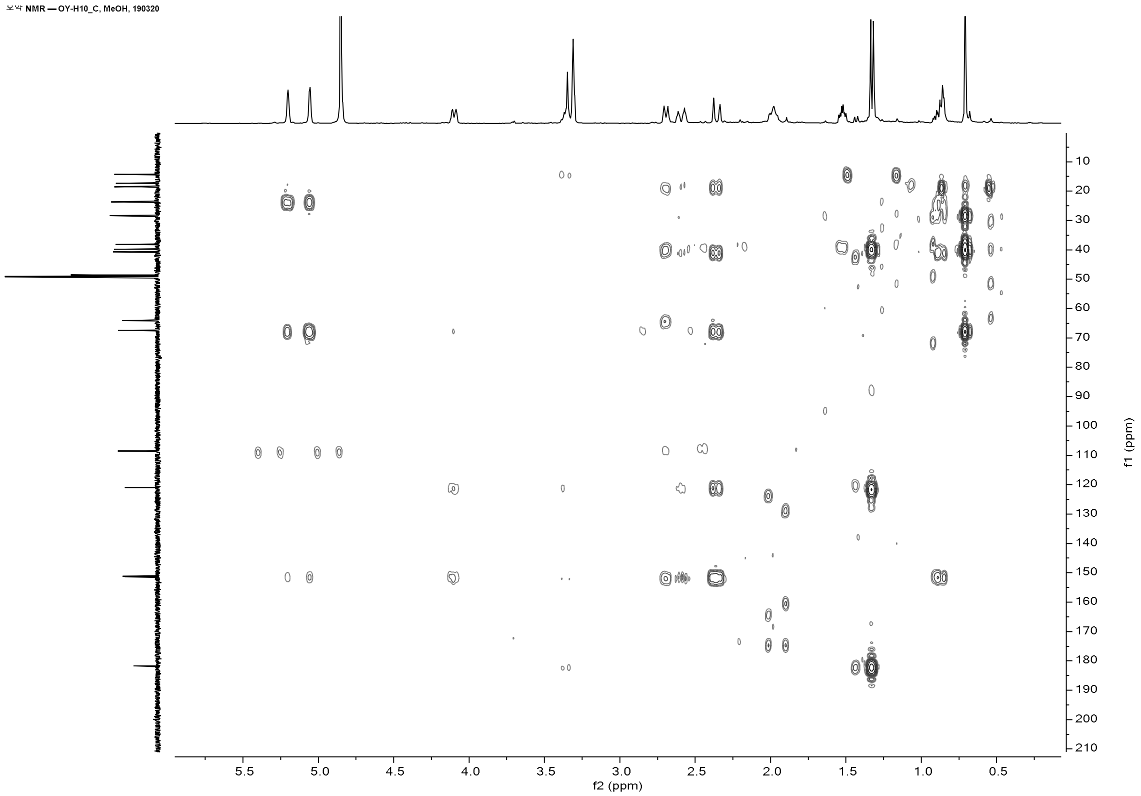


**Figure S17.** HMBC spectrum of **2** in $CD_3OD$.

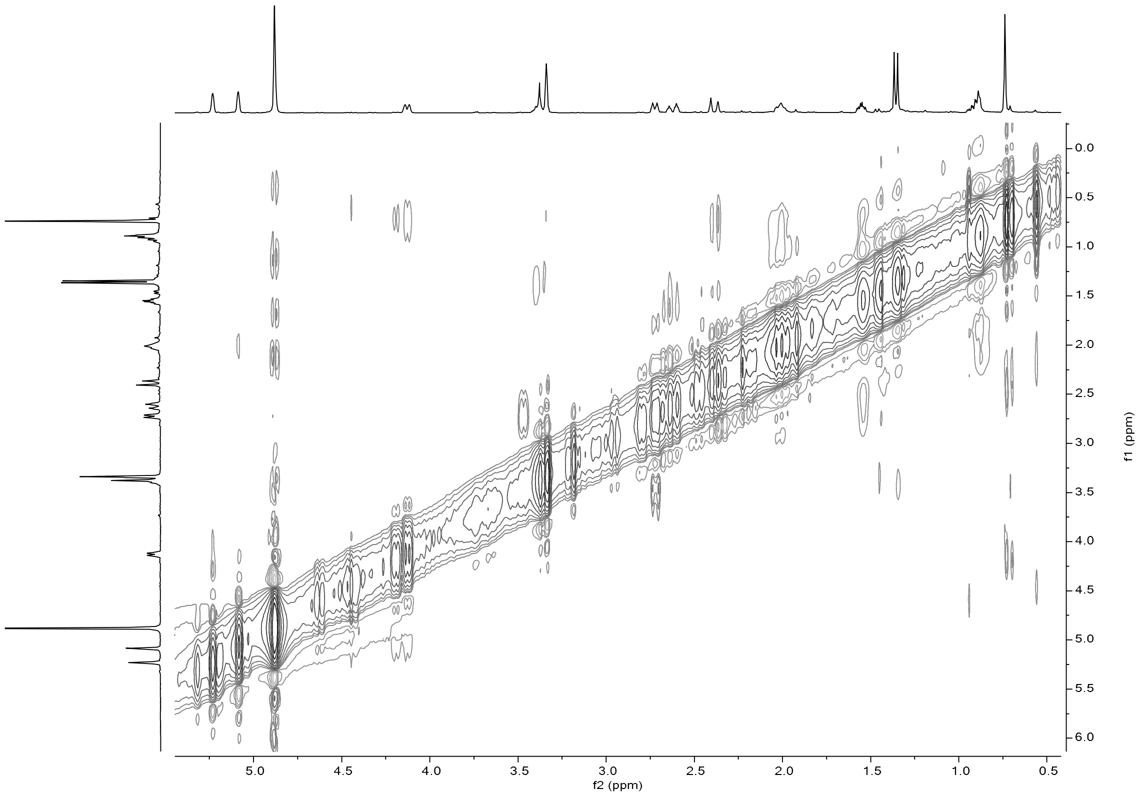


**Figure S18.** NEOSY spectrum of **2** in $CD_3OD$.

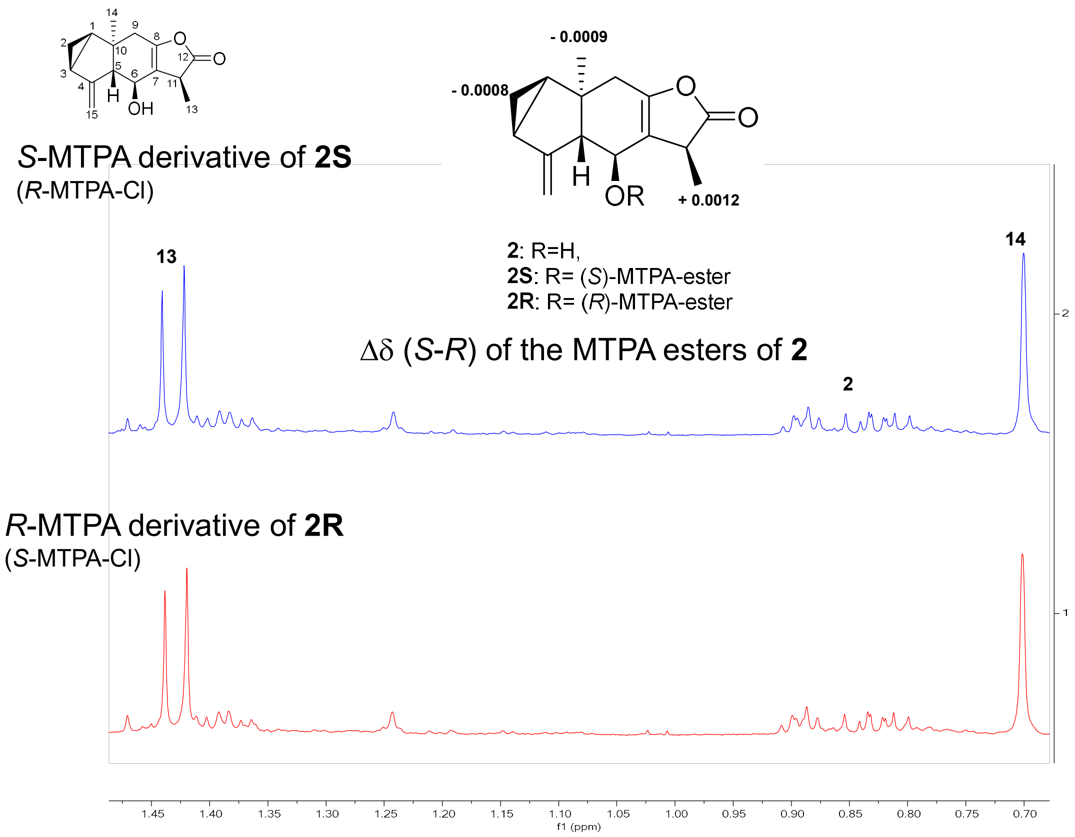


**Figure S19**. $^{1}$H NMR spectra of MTPA esters **2S** and **2R** and Δ*δ* (*S*-*R*) values for (*S*)- and (*R*)-MPTA esters (**2S** and **2R**).

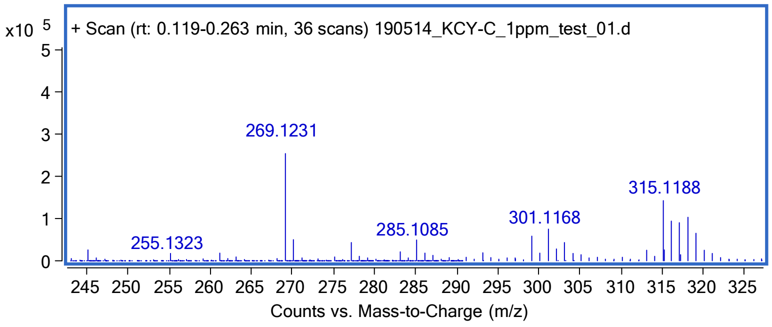


**Figure S20**. HRESI (qTOF) mass spectrum of **2**.
